# Light-induced hexatic state in a layered quantum material

**DOI:** 10.1038/s41563-023-01600-6

**Published:** 2023-07-06

**Authors:** Till Domröse, Thomas Danz, Sophie F. Schaible, Kai Rossnagel, Sergey V. Yalunin, Claus Ropers

**Affiliations:** 1 Department of Ultrafast Dynamics, Max Planck Institute for Multidisciplinary Sciences, Göttingen, Germany; 2 4th Physical Institute – Solids and Nanostructures, University of Göttingen, Göttingen, Germany; 3 Institute of Experimental and Applied Physics, Kiel University, Kiel, Germany; 4 Ruprecht Haensel Laboratory, Deutsches Elektronen-Synchrotron DESY, Hamburg, Germany

**Keywords:** Electronic devices, Topological defects, Characterization and analytical techniques, Phase transitions and critical phenomena, Structure of solids and liquids

## Abstract

The tunability of materials properties by light promises a wealth of future applications in energy conversion and information technology. Strongly correlated materials such as transition metal dichalcogenides offer optical control of electronic phases, charge ordering and interlayer correlations by photodoping. Here, we find the emergence of a transient hexatic state during the laser-induced transformation between two charge-density wave phases in a thin-film transition metal dichalcogenide, 1T-type tantalum disulfide (1T-TaS_2_). Introducing tilt-series ultrafast nanobeam electron diffraction, we reconstruct charge-density wave rocking curves at high momentum resolution. An intermittent suppression of three-dimensional structural correlations promotes a loss of in-plane translational order caused by a high density of unbound topological defects, characteristic of a hexatic intermediate. Our results demonstrate the merit of tomographic ultrafast structural probing in tracing coupled order parameters, heralding universal nanoscale access to laser-induced dimensionality control in functional heterostructures and devices.

## Abstract (web summary)

The authors report the emergence of a transient hexatic state during laser-induced transformation between two charge-density wave (CDW) phases in a thin film of the CDW material 1T-TaS_2_.

## Main

Collective behaviour beyond the single-particle picture fosters a variety of fundamental physical phenomena, such as superconductivity^1^ and ferromagnetism^2^, which involve the establishment of long-range order below a critical temperature. Due to the weaker screening and reduced phase space volumes for scattering, collective excitations are particularly important in low-dimensional systems. Prominently, plasmon and spin waves constitute elementary excitations of the one-dimensional Luttinger liquid^3^.

For ideal systems of reduced dimensionality^4^, the Mermin–Wagner–Hohenberg theorem prohibits long-range ordering at finite temperatures, owing to thermal fluctuations. Strictly speaking, this precludes symmetry-breaking phase transitions. Instead, the transition from a quasi-long-range-ordered, two-dimensional solid to a liquid phase is described within the framework of Kosterlitz–Thouless–Halperin–Nelson–Young (KTHNY) theory^5,6^. While the solid–liquid transition is of first order in three-dimensional systems^7^, the theory predicts two successive second-order transitions in two dimensions via a hexatic phase. This intermediate is characterized by intact orientational but reduced translational order arising from the presence of unbound topological defects^6,8^.

Experimental realizations of this transition have been actively pursued with two-dimensional model systems of colloidal spheres^8^, particles physisorbed on crystalline substrates^7^, skyrmion lattices^9^ and technologically relevant smectic liquid crystals^10^. Yet quasi-two-dimensional character is also readily found in layered materials^4^. Among those, transition metal dichalcogenides (TMDCs) stand out due to their highly anisotropic correlations^11^. This leads to the occurrence of direct-to-indirect bandgap transitions^12^, layer- and doping-dependent superconductivity^13–16^ and the formation of charge-density waves (CDWs)^17^. Due to the delicate interplay between the various degrees of freedom, the properties of these systems, often referred to as quantum materials^18^, are highly tunable by external stimuli^19–25^.

As a consequence, TMDCs susceptible to charge ordering often exhibit more than one thermodynamic CDW phase, governed by a varying balance of, for example, electron–phonon coupling, electronic correlations and Fermi surface nesting^17^. These phases may exhibit drastically different macroscopic properties, but only subtle changes of the periodic lattice distortion (PLD) coupled to the CDW^26^. As demonstrated in a wide range of ultrafast experiments, optical pulses can be used to transiently suppress CDW phases and drive transitions between them^26–40^, induce transient CDW order^41^ or open up paths into thermodynamically inaccessible hidden states^28,42–44^. The high degree of in-plane structural disorder induced by the optical excitation not only influences switching behaviour^36,38,45^, but also the final phase texture^37,42,43,46^.

The effective dimensionality of a CDW system is determined by the coupling between neighbouring layers. Hence, a phase transition that modifies interlayer correlations may lead to the occurrence of a dimensional crossover^47,48^. On ultrafast timescales, CDW stacking dynamics have recently been studied by means of ultrafast electron diffraction^34,39,40^ and X-ray diffraction^38,49^. In particular, an optically induced breakdown of excitonic correlations was shown to quench out-of-plane order^39^, and careful tuning of the photoexcitation density was used to realize interlayer phase boundaries with two-dimensional characteristics^50^—raising the question of if optical control may serve as a gateway to fundamentally low-dimensional phenomena such as the KTHNY transition.

In this Article, we report on the observation of a transient hexatic state obtained by optically quenching the room-temperature CDW phase of the TMDC 1T-TaS_2_. By means of ultrafast high-coherence nanobeam diffraction, we track the three-dimensional phase ordering following femtosecond optical excitation by analysing CDW diffraction spot shapes. At early times and prior to the establishment of the equilibrium stacking sequence, we identify a quasi-two-dimensional state characterized by a pronounced anisotropy in the CDW correlation function, indicative of an orientational preference of the CDW in the absence of translational symmetry. Based on time-dependent Ginzburg–Landau simulations, we conclude that this phenomenon parallels the predictions of KTHNY theory. Specifically, the kinetics of interacting topological point defects in a two-dimensional environment governs the transition between the two involved structural phases on ultrafast timescales.

## Ultrafast high-coherence nanobeam diffraction

Over the past two decades, ultrafast electron diffraction^51,52^ has evolved into a highly sensitive technique to probe structural phase transitions on femtosecond timescales^23,32–39,41^. State-of-the-art time-resolved electron diffraction instrumentation typically uses large-diameter beams (>100 μm spot size) for averaged probing of CDW amplitudes, addressing a compromise between a small spot size and sufficient reciprocal-space resolution.

**Fig. 1:**
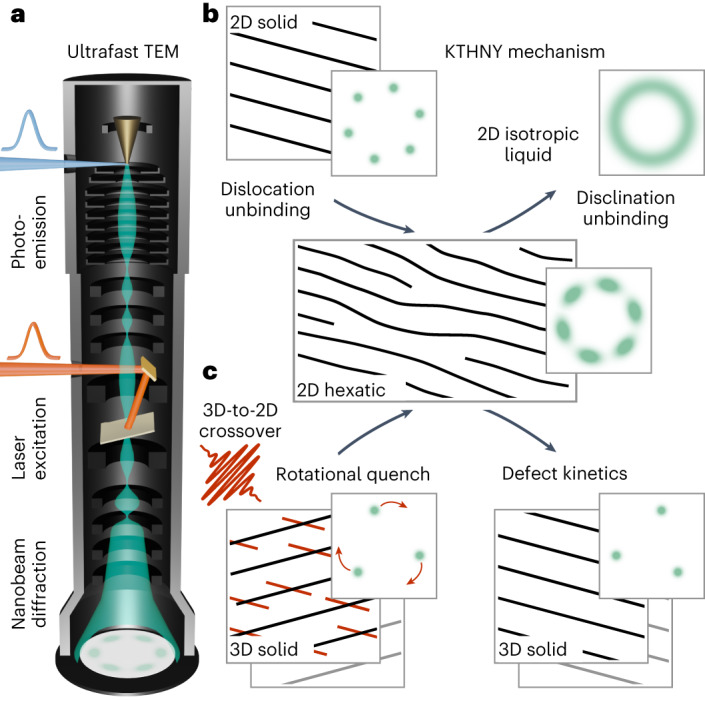
Transient hexatic state observed by high-coherence ultrafast nanobeam diffraction. **a**, Schematic of the Ultrafast TEM. A specimen is excited using femtosecond laser pulses (red). The resulting transient disorder is captured by ultrashort electron pulses (green) generated via linear photoemission (blue) from a field-emitter cathode. **b**, Microscopic mechanism of two-dimensional (2D) thermal melting via a hexatic phase, involving the step-wise unbinding of topological defects (KTHNY theory). Schematic diffractograms are shown in green. **c**, In the out-of-equilibrium scenario of the present work, hexatic order emerges as a result of the optical excitation of a three-dimensional (3D) solid, inducing a dimensional crossover and a spontaneous rotational break-up of the structure (red lines and arrows).

As the key experimental innovation of the present study, we specifically harness the capabilities of a laser-triggered field emitter in an ultrafast transmission electron microscope (UTEM)^53^ (Fig. 1a and Methods) to conduct high-coherence femtosecond electron diffraction. In particular, we combine high reciprocal-space resolution with an exceptionally narrow electron beam, in an extended series of eight beam tilts. The small effective source size of our set-up results in a transverse coherence length of up to one-tenth of the beam diameter, enabling a precise measurement of in-plane spot profiles and allowing us to distinguish phases characterized by similar periodicities (Methods). Simultaneously, the nanometric probe beam guarantees diffraction from a sample region of sufficient homogeneity in terms of thickness and orientation, as required for the quantitative investigation of stacking dynamics. The fingerprint of such structural modifications is often encoded in low-intensity diffracted signals. We increase the sensitivity to these features by means of a sample design tailored to drive the transformation at an unprecedented laser repetition rate of 610 kHz, maximizing the duty cycle of our measurement scheme (Methods).

**Fig. 2:**
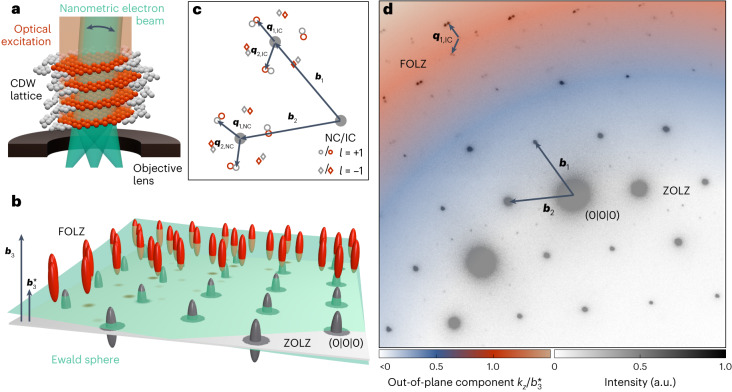
Reciprocal lattice of 1T-TaS_2_ and diffraction geometry. **a**, Visualization of the 1T-TaS_2_ specimen located within the objective lens of the UTEM. The optical excitation drives a transformation of the layered CDW lattice between two incommensurate CDW phases (grey and red spheres), probed by a nanometre-sized electron beam of variable tilt (double arrow). **b**, Ewald construction visualizing the diffraction geometry. The intensity scattered into individual first-order CDW spots is governed by proximity to the Laue condition, that is, where the Ewald sphere (green) intersects the CDW reciprocal lattice rods (red). Accessing the FOLZ requires an electron beam tilted slightly away from the [001] zone axis. The low curvature of the Ewald sphere for 120 keV electron energy results in a dense sampling of the rod shape. **c**, Reciprocal lattice of the NC phase (open grey) and IC phase (open red) of 1T-TaS_2_ (first-order spots only). Due to the CDW stacking periodicity, the CDW spots are located in the FOLZ (*l* = 3*n* ± 1 in equation (1)) of the CDW lattice, while the related host lattice reflections (solid grey) lie in the ZOLZ. **d**, Temporally averaged experimental diffractogram. The high electron beam coherence leads to a clear separation of NC and IC spots. The coloured overlay indicates the local height *k*_*z*_ of the Ewald sphere above the ZOLZ in units of *b*_3_*. While first-order spots of both phases are the dominant feature in the FOLZ and contain information on the stacking periodicity, the low-intensity second-order NC spots appearing between bright host reflections in the ZOLZ are a sensitive measure of the CDW amplitude.

In the experiments (Fig. 2a), we excite a free-standing 70 nm thin film of the prototypical CDW material 1T-TaS_2_ at room temperature, using ultrashort laser pulses (800 nm wavelength, 150 fs duration, between 0.3 mJ cm^−2^ and 7.0 mJ cm^−2^ fluence). As a function of a variable temporal delay Δ*t*, we capture the transient distribution of CDW spot intensities and profiles using high-coherence ultrashort electron pulses (120 keV beam energy, 500 fs duration, between 170 nm and 1.5 μm spot size, below 0.1 mrad convergence semi-angle).

## Incommensurate CDW phases in 1T-TaS_2_

We investigate the photoinduced transition between two incommensurate CDW superstructures in 1T-TaS_2_ (grey and red regions in Fig. 2a). In thermal equilibrium and at temperatures above 353 K, the in-plane modulation wave vectors **q**_*i*,IC_ (with *i* ∈ {1, 2}) of the CDW/PLD are aligned along the lattice vectors **a**_*i*_ of the hexagonal host^54^. Due to the presence of a gapless long-range phase fluctuation (or ‘phason’) mode, the CDW in this high-temperature incommensurate (IC) phase is effectively free-floating and only weakly coupled to neighbouring layers^55^. As such, the IC phase in 1T-TaS_2_ is an ideal host for topological defects and, potentially, hexatic ordering^56^.

Below the phase transition temperature, the modulation remains incommensurate and the wave vectors **q**_*i*,NC_ form an angle of ~12° with the lattice vectors **a**_*i*_ (the nearly commensurate (NC) phase). With substantial contributions from higher harmonics, the real-space structure exhibits a network of discommensurations separated by domains of commensurate character in which the CDW/PLD is locally locked-in with the crystal structure^54^ (compare with Supplementary Fig. 3). Despite their different in-plane structures, both phases exhibit a commensurate, threefold stacking periodicity along the out-of-plane direction (**a**_3_* = 3**a**_3_) (ref. ^54^).

The reciprocal lattice of the host material is given by basis vectors **b**_*i*_. The modulation wave vectors **q**_*i*_ span the reciprocal CDW lattice (*m*_1_, *m*_2_) around every individual main lattice point (*h*, *k*, *l*; Fig. 2c). Hence, each lattice point **k** can be identified by^54^

1 $$\begin{array}{r}{\bf{k}}=h\,{\bf{b}}_{1}+k\,{\bf{b}}_{2}+l\,{\bf{b}}_{3}^{* }+{m}_{1}\,{\bf{q}}_{1}+{m}_{2}\,{\bf{q}}_{2}.\end{array}$$

This notation absorbs the commensurate out-of-plane component of the CDW in a supercell, effectively leading to a shorter reciprocal lattice vector **b**_3_* = **b**_3_/3 and certain systematic absences in diffraction experiments. Specifically, whereas all main reflections (with *m*_1_, *m*_2_ = 0) lie in planes *l* = 3*n* (where *n* is an integer), first-order CDW spots with (*m*_1_, *m*_2_) = (1, 0), (0, 1) and (−1, 1) (and those with opposite sign) are located in planes *l* = 3*n* ± 1 with non-vanishing out-of-plane wave vector component *k*_*z*_. Thus, they appear only upon tilting the electron beam away from the [001] zone axis^34,54^.

A visualization of the corresponding diffraction geometry is shown in Fig. 2b. Under tilted-beam conditions, electron diffractograms typically feature spots in more than one Laue zone (Fig. 2d). Main reflections are located in the zero-order Laue zone (ZOLZ) close to the unscattered beam and appear bright compared to the second-order CDW spots surrounding them. As a result of the CDW stacking sequence, first-order CDW reflections are found in the first-order Laue zone (FOLZ) and at larger wave vectors for moderate beam tilts (Supplementary Videos 1 and 2 and Supplementary Fig. 3).

## Dynamics of the in-plane correlation length

**Fig. 3:**
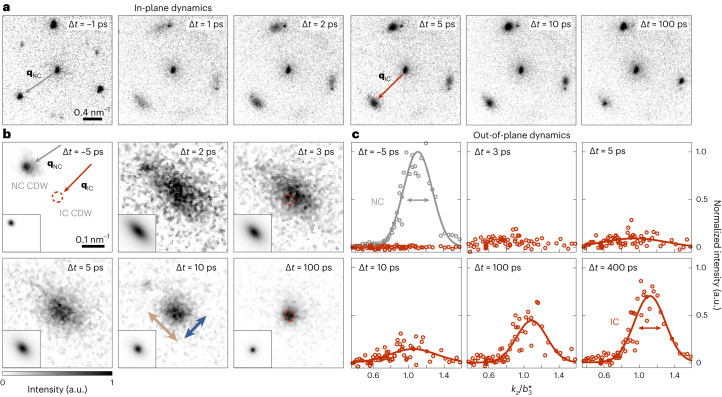
Temporal evolution of the in-plane and out-of-plane CDW spot shapes. **a**, Section of reciprocal space in the FOLZ at representative temporal delays Δ*t*. A triplet of first-order CDW diffraction spots surrounds the associated structural reflection; satellites with lower intensities are higher-order spots of the NC phase attributed to neighbouring reflections. **b**, Average over all visible first-order CDW diffraction spots close to the FOLZ. The equilibrium IC spot position is given by dashed red circles. Brown and blue arrows indicate the evaluation axes for the azimuthal and radial spot widths, respectively. The insets show a two-dimensional pseudo-Voigt fit of the spot shape. **c**, Out-of-plane spot profiles at representative temporal delays Δ*t* reconstructed from in-plane spot intensities measured at different *k*_*z*_ (red circles). The scaled equilibrium spot profile of the NC phase before time zero is displayed for reference (grey circles). The solid lines represent Gaussian fits of the rocking curves. The spot widths of the NC phase before time zero and of the IC phase at 400 ps are indicated by arrows and amount to 0.23 nm^−1^ (full-width at half-maximum) each.

Figure 3a shows a series of time-resolved diffraction patterns, averaged over several main reflections in the FOLZ (2.8 mJ cm^−2^ pump fluence, 1.1 μm electron spot diameter). Before optical excitation (that is, time zero), we observe a triplet of sharp first-order spots of the initial NC phase. The additional low-intensity reflections in between stem from higher CDW orders (>2). After excitation, the NC spot intensity is largely suppressed within 1 ps, followed by the emergence of nearby reflections with (*m*_1_, *m*_2_) = (1, 0), (0, 1) and (−1, 1), which are evidence of the nascent IC phase and which exhibit a pronounced anisotropic broadening^35,36,57^, as well as a few-picosecond increase in intensity^33^ (also compare with Le Guyader et al.^34^). Additionally, a slight increase of scattered intensity is detected at (*m*_1_, *m*_2_) = (−1, 0), (0, −1) and (1, −1), that is, opposite of the bright IC spots. Only after these early-stage dynamics do the spot shapes become isotropic, and solely the bright IC spots remain (as in the image at 100 ps).

**Fig. 4:**
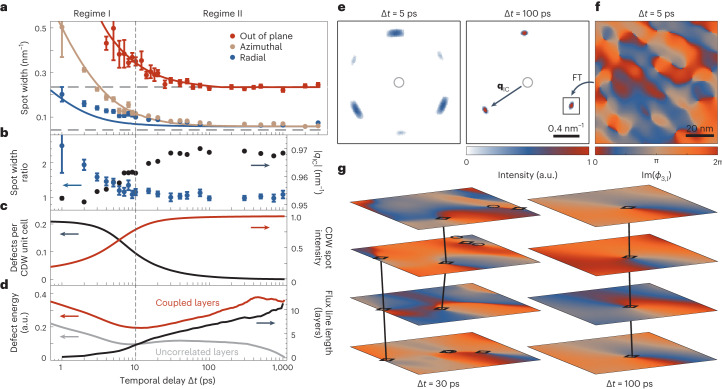
Dynamics of the IC spot profile and simulation results. **a**, Azimuthal (brown) and radial (blue) IC spot widths (full-width at half-maximum) extracted from the in-plane data shown in Fig. 3b (see brown and blue arrows at 10 ps delay) and out-of-plane IC spot widths (red) derived from the reconstructed rocking curves shown in Fig. 3c. All curves approach the NC equilibrium spot widths at late delay times (dashed grey lines) when the structural correlation length exceeds the reciprocal space resolution in the experiments. The depicted data points and error bars represent the results and the corresponding 68% confidence intervals from the fitting of the spot profile as described in Fig. 3. Solid lines represent the corresponding temporal evolution of the spot widths in the simulation corrected for the instrument resolution (Methods). **b**, The left axis shows the temporal evolution of the ratio between azimuthal and radial spot width (blue). The right axis shows the dynamics of the CDW wave number |*q*_IC_| extracted from the fits in Fig. 3b (black). Similar to **a**, the depicted values and error bars are the results of fits to the temporal evolution of the spot profile. **c**, The left axis shows the density of CDW phase vortices in the simulation (black). The right axis shows the temporal evolution of the simulated CDW intensity (red). **d**, The left axis shows the simulated energy per phase vortex for a stack of correlated (red) and uncorrelated (grey) layers. The right axis shows the average flux line length corrected for the influence of the coincidental alignment present at early times (Methods). **e**, Example simulated diffractograms in a section of reciprocal space as in Fig. 3a. In accordance with the experimental results, we find six azimuthally broadened diffraction spots in the FOLZ at early times, while only three CDW reflections indicative of the three-dimensional CDW are present at late times. **f**, Simulated phase modulation Im(*ϕ*_3,*l*_) within an individual layer, related to the phase pattern by a Fourier transformation (FT). Note the anisotropic distortions in the vicinity of neighbouring phase singularities that induce the azimuthal broadening in the CDW correlation function for high defect densities. **g**, Three-dimensional CDW phase modulation Im(*ϕ*_3,*l*_) in 4 of the 51 simulated layers. The stacking sequence favours a local phase shift of 2π/3 between adjacent layers and the alignment of phase vortices into ‘flux lines’ (marked as black circles and diamonds for opposite chirality). The images display the same region as in **f**.

For a quantitative analysis, we recorded a second image series with a longer camera length, that is, with optimized reciprocal-space resolution (Fig. 3b; 3.7 mJ cm^−2^ pump fluence, 1.1 μm electron spot diameter; also Supplementary Videos 3 and 4). We fitted the spot shape at every temporal delay (as shown in the insets) and extracted the azimuthal and radial spot widths (indicated by brown and blue arrows, respectively). The results are shown in Fig. 4a (brown and blue data points). From a spot width ratio of >2 shortly after optical excitation (blue circles in Fig. 4b), the reflections assume an isotropic, yet broadened shape within 10 ps (temporal regime I). On longer timescales, the IC spot width approaches that of the NC peak measured before time zero (temporal regime II; corresponding dashed grey line in Fig. 4a). Notably, this behaviour coincides with a monotonous growth of the IC wave vector, which initially is ~2% shorter than in the equilibrium phase at late delay times (black circles in Fig. 4b).

The different spot profiles along the azimuthal and radial directions (in relation to the nearby main reflection) in temporal regime I are a hallmark of hexatic order^6^ (Fig. 1b), suggesting that the optical excitation induces transient two-dimensional behaviour in 1T-TaS_2_ on ultrafast timescales. To test this hypothesis, we probed the effective dimensionality of the system, analysing the intensities of reflections with different out-of-plane momenta.

## Reconstruction of the CDW rocking curve

Under tilted-beam conditions, the lattice rod is densely sampled across the various spots present in the FOLZ of every single diffractogram (visualization in Fig. 2b and colour overlay in Fig. 2d for the experimental tilt angle of ~2.4°). To further enhance the sensitivity and fully cover reciprocal space, we performed delay scans with eight different beam tilts. Correlating the scattered intensities before time zero with dynamical diffraction simulations (Supplementary Notes 1 and 2), we determined the tilt angle for any of these scans with an accuracy of better than 0.2° (Methods and Supplementary Fig. 1).

**Extended Data Fig. 1:**
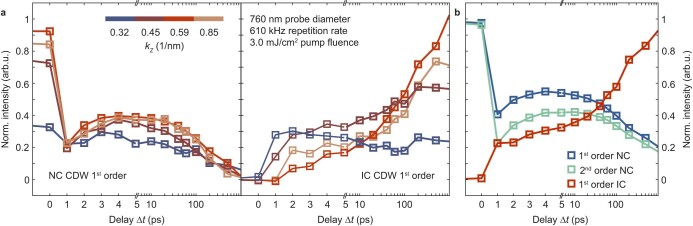
CDW diffracted intensity for the reconstruction of rocking curves. **(a)** Intensity of first-order NC (left) and IC (right) diffraction spots as a function of temporal delay for the data set shown in Fig. 3c in the main text. The curves are additionally sorted and binned by the k_z_-component of the respective reflections. The scattered intensity at the IC spot position before time zero is subtracted for both types of curves as a measure of the inelastic background in the images. The NC curves are normalised with respect to the measured intensity before time zero and show similar behaviour as a function of temporal delay. For the IC curves, we normalise to the measured intensity after 300 ps. During the phase formation, IC spots probed at a k_z_ -component closer to the ZOLZ display an initial overshoot (blue curve). In contrast, reflections in the FOLZ are characterised by a continuous intensity increase (red curve), leading to the sharpening of the reciprocal lattice rod along the out-of-plane direction described in the main text and Fig. 3. **(b)** Average intensity of first-order NC (blue), first-order IC (red) and second-order NC spots (green). Curve normalisation as described in **(a)**.

Sorting the scattered intensities of spots with comparable CDW structure factors by their associated out-of-plane components *k*_*z*_, we reconstructed the CDW rocking curve from 11 individual IC reflections with a total of 72 values of *k*_*z*_ throughout our tilt series at every stage of the dynamics (Fig. 3c and also Methods; 3.0 mJ cm^−2^ pump fluence, 760 nm electron spot diameter). At early times, the intensity increases independently of *k*_*z*_, indicating a pronounced elongation of the reciprocal lattice rods along the **b**_3_* direction (Fig. 3c at 3 ps and Extended Data Fig. 1). This implies an initial loss of long-range structural coherence along the out-of-plane direction, resulting in a stack of uncorrelated two-dimensional CDW layers. Subsequently, a well-defined spot profile emerges and narrows until approaching that of the three-dimensionally stacked NC phase measured before time zero (red data points and asymptotic dashed grey line in Fig. 4a).

## Transient hexatic order induced by topological defects

The experimentally observed diffraction spot patterns strikingly resemble those of hexatic phases realized in other low-dimensional materials^7,10,56^ and mesoscale systems^8,9^ (Fig. 1b). The associated suppression of translational symmetry is apparent from an overall in-plane broadening of the related diffraction features, specifically, a diffuse spot shape along both the radial and the azimuthal directions. Distinct from the closed diffraction ring observed for an isotropic liquid that possesses only short-range correlations, however, the modulation of the hexatic structure factor along the azimuthal direction indicates an orientational preference, that is, a weakly intact sixfold bond orientational order. In the framework of KTHNY theory, the transition from a crystal into such an anisotropic fluid^5^ is described in terms of the material’s elastic moduli^6^. At the critical point, the divergence of the elastic constant of the solid leads to the unbinding of dislocation pairs, enhancing the anisotropy of the structure factor beyond the value determined by the material’s Poisson ratio^58^. While translational correlations decay exponentially, the stiffness against a rotation of the bond-angle field, that is Frank’s constant, remains finite. As a result, the orientational order exhibits an algebraic decay, and the spot width ratio not only depends on the mean dislocation distance, but also scales with the system size^58^.

**Extended Data Fig. 2:**
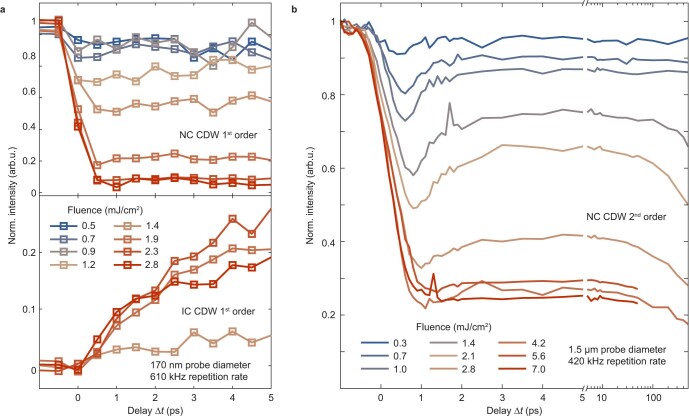
Ultrafast nanobeam diffraction of CDW dynamics. **(a)** Intensity of first-order NC (top) and IC (bottom) diffraction spots as a function of temporal delay for varying pump fluence. The data was recorded under tilted-sample conditions with an electron beam size of 170 nm and a repetition rate of 610 kHz. The NC curves are normalised as in Extended Data Fig. 1. We observe an initial suppression within 500 fs that scales with the pump fluence. Above a threshold of around 2 mJ/cm^2^, the measured NC intensity after time zero equals the inelastically scattered background. For such higher fluences, we measure an increase of IC spot intensity within 5 ps with a temporal characteristic independent of the pump fluence. The IC curves are normalised to the corresponding NC curve intensities measured before time zero. **(b)** Delay scans of second-order NC intensity for varying pump fluence with the sample tilted into the zone-axis. In contrast to **(a)**, the electron beam has a larger diameter (1.5μm), adding a spatiotemporal component to the dynamics following the initial suppression. For intermediate fluences, we witness a partial recovery of NC intensity after around 5 ps, followed by a second suppression at later delays. We attribute this behaviour to a global vibration of the specimen on the timescale of 500 ps and beyond, that modulates the detected intensity of diffraction spots in the ZOLZ as a result of the changing relative angle of incidence of the electron beam onto the sample. Furthermore, the locally varying excitation density leads to the formation and subsequent growth of IC phase domains on longer timescales (see Methods).

In the following, going beyond phenomenological similarities, we argue that closely related microscopic mechanisms responsible for the formation of the intermediate thermodynamic phase may also govern non-equilibrium dynamics involving topological disorder, justifying the notion of a transient hexatic state. The two CDW modifications involved in the experiment are not commensurate with each other. As a result, there is no continuous global deformation of the crystal during the phase transformation. Instead, photoexcitation gives rise to a spontaneous break-up of the system by inducing a sudden rotation of the equilibrium CDW wave vectors (Fig. 1c). The stress within the structure, assumed to follow from the spatially varying overlap of the PLD’s of both phases^33^, is compensated by the formation of topological defects, apparent from the broadening of IC diffraction spots directly after time zero. So far, there is no direct experimental evidence that the nascent density or real-space distribution of such defects is determined by the specifics of the NC phase. However, one may speculate that the spatial beating period of the two textures, represented by a moiré pattern, may limit the initial correlation length of the hexatic state. The fact that the initial broadening of the emerging peaks is of a similar order as the angular separation of equilibrium IC and NC spots may give some indication in support of this hypothesis. Such a primarily geometrical interpretation would also be consistent with the observed negligible dependence of the dynamics on the pump laser fluence above the phase transition threshold^36^ (Fig. 3 and Extended Data Fig. 2a). Yet, the emergence of the IC phase occurs only after a complete suppression of the NC spots, and there is no local phase coexistence. Remnants of the NC phase present at low and intermediate pump fluences are spatially separated from the hexatic state and show no signs of laser-induced disorder. Thus, further work may explore whether there is a deterministic component in the creation mechanism, in addition to a spontaneous break-up resulting in a random distribution of defects.

In the past, both CDW dislocations^36–38,57^ and domain walls^35,38^ have been invoked to describe the transiently disordered structure on a microscopic scale. While CDW states in 1T-TaS_2_ are known to support both types of topological features^43^, in our measurements, conceptual agreement between the early stages of the IC phase formation kinetics and KTHNY behaviour is further substantiated by the temporal evolution of the IC wave vector (black circles in Fig. 4b). The observed initial shortening has recently been attributed to a dislocation-induced self-doping of the electronic system^38^. The concurrent highly uncorrelated out-of-plane structure that facilitates intrinsically two-dimensional behaviour is apparent from the elongation of reciprocal lattice rods (Figs. 3c and 4a), also giving rise to the simultaneous visibility of all six IC reflections in the same Laue zone (Fig. 3a; 1 ps to 5 ps).

To further explore how the kinetics of topological defects govern the three-dimensional ordering after a sudden quench of the system, we implement a multilayer time-dependent Ginzburg–Landau simulation. The underlying free-energy functional is derived from Nakanishi’s model describing the most important CDW phases in 1T-TaS_2_ and their out-of-plane configurations (Methods for a more detailed description). We express the IC modulation in terms of three coupled order parameters *ϕ*_*j*,*l*_ corresponding to the directions of the three in-plane modulation wave vectors **q**_*j*,IC_ in the layer *l* of the atomic structure. The physical charge-density modulation *ρ(****r****)* (***r:*** three-dimensional position) (Supplementary Fig. 2) is then given by

2 $${\rho }_{l}({\bf{r}})={{{\rm{Re}}}}\left[\mathop{\sum}\limits_{j}\exp \left(2\uppi i\,{\bf{q}}_{j}\cdot \bf{r}\right){\phi }_{j,l}\right].$$

This model is capable of closely reproducing the experimentally observed temporal evolution of the reciprocal lattice rod (solid lines in Fig. 4a) and the CDW diffraction spot intensities above the NC-to-IC transition temperature (Extended Data Figs. 1b and 2a). At early times, nonlinearities in the free-energy landscape drive a local build-up of the IC amplitude (red curve in Fig. 4c) without long-range correlations, a build-up that unfolds independently within the individual layers. Due to the reduced dimensionality and the negligible lock-in of the incommensurate CDW with the underlying atomic lattice, a high density of uncorrelated phase singularities constitutes the most favourable configuration for this disordered initial state. The pairing of these point defects with opposite chirality in the phases of two of the three order parameters then leads to the formation of physical CDW dislocations. Subsequently, gradient terms govern the establishment of a long-range-ordered IC phase via defect kinetics and recombination, discriminating between a radial and an azimuthal stiffness of the CDW that shapes the simulated diffraction spot anisotropy throughout the entire dynamics (parameters *u* and *v*, respectively, in equation (6) in the Methods).

The set of parameters that best reproduces our experimental observations yields a transition from high to low defect density after 10 ps (black curve in Fig. 4c), coinciding with the observed temporal evolution of the IC wave vector and the overall sharpening of diffraction spots in temporal regime I (solid lines in Fig. 4a,c). The large concentration of dislocations not only limits the long-range coherence of the CDW, but also reproduces the characteristic shape of the hexatic structure factor, as the distortion of the CDW phase pattern is particularly enhanced and anisotropic in the vicinity of closely neighbouring defects (Fig. 4f; also Supplementary Video 5). Based thereon, we attribute the dynamics within the first 10 ps of the phase formation to transient KTHNY behaviour, invoked by the interaction of unbound dislocations and supported by a temporary loss of interlayer correlations. Accordingly, the evolution of the average free energy associated with an individual phase vortex parallels that of a purely two-dimensional system, as seen for a reference simulation without interlayer coupling (red and grey curves in Fig. 4d).

Beyond temporal delays of 10 ps and after the spot shape anisotropy has decayed, we observe the establishment of long-range crystalline order and the onset of three-dimensional behaviour. In contrast to the two-dimensional reference system, the energy of individual vortices increases, and their resulting correlation in adjacent layers leads to the formation of ‘flux lines’ (black lines in Fig. 4d,g; Methods for a more detailed description). Similarly, interlayer coupling in liquid crystals has been shown to suppress the fluctuations in the bond orientational order parameter, thereby reducing the angular width of the diffraction spots^10^. We therefore believe that the observed loss of stacking order at early times is a prerequisite for inducing the intrinsically two-dimensional hexatic intermediate in 1T-TaS_2_. It is intriguing to speculate whether such a state could in fact be realized as a thermodynamically stable phase during the metallic-to-IC transition in a 1T-TaS_2_ monolayer.

## Outlook

In conclusion, the identification of hexatic order and stacking dynamics in our study is enabled by ultrafast diffraction with a nanoprobe of exceptional collimation. These results tie in with recent observations of laser-induced transient phases^30^ and dimensional crossovers^39,50^, exemplifying how optical control over interlayer correlations can be used to host low-dimensional states and transitions. Addressing recurring experimental challenges in ultrafast and materials science, this approach promises to advance high-resolution non-equilibrium investigations in systems characterized by weak structural signatures, submicrometre sample sizes and considerable spatial heterogeneity^59^. As such, nanoscale structural analysis will guide the design of applications harvesting laser-induced functionality by material composition and tailored responses to external stimuli.

## Methods

### Ultrafast nanobeam diffraction experiments

Ultrafast electron diffraction^51,52^ allows for the study of phase transitions^23,32–39,41,57^ and transient phonon populations^60–64^ on ultrafast timescales. Conducting such experiments in an Ultrafast TEM (Fig. 1a)^53,65–70^ extends these capabilities by the versatility of electron microscopy in terms of beam shaping and a wide range of additional measurement schemes within the same instrument^31,40,70–72^. In particular, laser-triggered field emitters yield UTEM beams of enhanced transverse coherence^53,67,68^.

The Göttingen UTEM is based on a JEOL JEM-2100F transmission electron microscope modified to enable the investigation of ultrafast dynamics. Femtosecond laser pulses (515 nm wavelength after frequency doubling of the output of a Light Conversion PHAROS femtosecond laser, 610 kHz repetition rate) are used to generate ultrashort electron pulses from the microscope’s ZrO/W Schottky emitter via linear photoemission. A fraction of the laser output is converted to 800 nm wavelength by optical parametric amplification (Light Conversion ORPHEUS-F) and is incident on the sample (p-polarized) at a variable temporal delay with respect to the electron pulses. The reciprocal-space resolution in our measurements under collimated illumination was limited by the degree of coherence of the electron beam. We find a value of up to 10%, linking the beam diameter in the sample plane to the transverse coherence length that determines the minimum width of CDW diffraction spots. For the data displayed in Fig. 3a,b, for example, this brings about a resolution of 0.057 nm^−1^ (full-width at half-maximum; Fig. 4a), indicative of a coherence length of 18 nm for a beam diameter of 1.1 μm. A similar calculation for the data displayed in Supplementary Fig. 3a yields a degree of coherence of 9%, resulting in a transverse coherence length of 15 nm for a beam diameter of 170 nm. Further technical details on the instrumentation are given in ref. ^53^.

Snapshots of the non-equilibrium dynamics are recorded on a direct electron detection camera (Direct Electron DE-16) and processed by an electron counting algorithm. The diffractograms presented in this Article have been integrated for 3 min (Fig. 3a), 7.5 min (Fig. 3b) and 5 min (Figs. 2d and 3c) per temporal delay.

### Reconstruction and fitting of CDW rocking curves

As described in the main text, the out-of-plane component of the reciprocal lattice rod *k*_*z*_ is accessible by recording diffractograms under a relative orientation between the sample and probe beam illumination that deviates from the [001] zone axis^73^. In our experiments, we reconstruct the three-dimensional CDW reciprocal lattice rod by tilting the electron beam, assigning the respective *k*_*z*_ component to the measured intensity of every first-order CDW diffraction spot in the individual images of our beam tilt series. In addition to a modulation by *k*_*z*_, the intensity of a CDW spot of order *n* at a scattering vector **k** depends on the structure factor *V(****k****)*, which can be expressed in terms of a Bessel function of the first kind, *J*_*n*_(*x*) (ref. ^55^):

3 $$V({\bf{k}})\propto {J}_{n}(2\uppi \,{\bf{k}}\cdot {\bf{A}}).$$

For the longitudinal distortion with comparably small amplitude **A**_*i*_ found here (**A**_*i*_ ∥ **q**_*i*_, where **q**_*i*_ is the CDW wave vector)^74^ and considering high-energy electrons, the diffractograms mainly contain information about the modulation of the tantalum sublattice^75^. By comparison, sensitivity to the periodic distortions of the sulphur atoms would be enhanced in a backscattering geometry with low-energy electrons^76^. Additionally, CDW spots, to a good approximation, can be sorted by their respective scattered intensity into three groups such that the CDW rocking curve can be reconstructed from multiple CDW spots within a single image (Supplementary Fig. 1d–f).

For the CDW rocking curves displayed in the main text, we consider only the brightest reflections, where the angle *φ* between **k** and **q**_*i*_ (Supplementary Fig. 1b) is smaller than 45° (Supplementary Fig. 1d for the resulting NC rocking curve at Δ*t* < 0 ps). By comparison, CDW reflections with *φ* > 45° are less intense and more heavily affected by dynamical scattering effects (Supplementary Fig. 1e,f). The reconstruction of the CDW rocking curves is based on the *k*_*z*_-dependent temporal evolution of scattered CDW intensities, shown in Extended Data Fig. 1. Following optical excitation, the NC CDW amplitude is suppressed within 1 ps irrespective of the out-of-plane component. By contrast, IC diffraction spots situated at *k*_*z*_ components deviating from the FOLZ display an initial intensity overshoot, whereas those spots probed directly in the FOLZ experience a monotonous increase of scattered intensity over the entire temporal regime (Extended Data Fig. 1a).

The length of the reciprocal lattice rod at every temporal delay is determined by fitting a Gaussian-shaped profile to the rocking curves inferred from these delay curves. Every rocking curve consists of intensities scattered into 11 individual IC reflections probed at varying *k*_*z*_ values in our tilt series, resulting in a total of 72 out-of-plane momenta considered for the fitting of the rod shape. The majority of these CDW reflections lie at out-of-plane wave vector components smaller than the equilibrium stacking periodicity of the CDW. To enhance the fitting weight of the brightest spots close to the centre of the rocking curve, we linearly interpolate the measured CDW intensity to gain a homogeneously spaced distribution along *k*_*z*_. The fit results with the corresponding error bars are displayed in Figs. 3c and 4a in the main text.

### Specimen preparation and fluence-dependent CDW dynamics

The investigated 70 nm thin film of 1T-TaS_2_ has been obtained by ultramicrotomy. Details on the preparation process and a comprehensive characterization of the specimen can be found in the supplementary information of ref. ^31^. The sample design has been optimized to allow the driving of laser-induced dynamics with high repetition rates (up to 610 kHz for the experiments shown in the main text), enhancing the sensitivity to low-intensity features in diffraction and imaging. The 1T-TaS_2_ thin film is suspended below a gold aperture with a diameter of ~2 μm in order to confine the effective excitation volume to the area within the gold mask. Additionally, the gold film serves as a heat bath for energy dissipation via thermal diffusion, and the design ensures that the shadowed parts of the 1T-TaS_2_ remain in the NC phase throughout the entire dynamics, prompting a recrystallization of the same NC chirality at the end of every pump–probe cycle.

The enhanced duty cycle in our measurements allows us to further limit the diameter of the collimated electron beam by apertures down to 170 nm in the specimen plane. The resulting delay curves of first-order and second-order CDW spots are shown in Extended Data Fig. 2a,b, respectively. Following the optical excitation, the intensity scattered into both first-order and second-order NC CDW spots is suppressed within 1 ps. After the initial quench, we observe a partial recovery of NC intensity within 5 ps for fluences below the phase transition threshold, indicative of the typical reshaping of the free-energy landscape due to the thermal equilibration of the electronic subsystem and the lattice^77^. On longer timescales, energy dissipation unfolds laterally within the layers via thermal diffusion^31^, leading to a second suppression of NC spot intensity after 100 ps. The initial NC phase is then re-established after a few nanoseconds. Overall vibrations of the 1T-TaS_2_ membrane on similarly slow timescales change the relative angular orientation between the sample and the incident electron beam, additionally modulating scattered intensities, particularly under tilted illumination (Extended Data Fig. 1).

For fluences above the phase transition threshold of ~2 mJ cm^−2^, IC CDW spots emerge within the first few picoseconds of the dynamics. We observe a negligible influence of the IC amplitude build-up on the laser pump fluence (Extended Data Fig. 2a). Ultrafast dark-field imaging of the phase transition further suggests instant phase switching throughout the entire stack of layers^31^.

### Origin of remnant NC diffraction spots

The sample design described above results in a spatially heterogeneous laser excitation of the 1T-TaS_2_ specimen due to scattering effects of the 800 nm laser excitation within the 2 μm gold aperture^31^. At intermediate pump fluences, the associated interference pattern will be composed of weakly and strongly pumped regions, resulting in the emergence of IC domains separated by areas with an only partially suppressed NC phase. The ultrafast electron diffraction averages over this spatial heterogeneity, which leads to the presence of both types of diffraction spots for intermediate fluences and electron beam diameters larger than a few hundred nanometres (also Extended Data Fig. 2). Along the out-of-plane direction, the phase transition unfolds uniformly within the IC domains^31^, such that there is no contribution from averaging or phase competition along the beam direction. Moreover, the overall influence of phase boundaries on the shape of the diffraction spots is negligible.

### Time-dependent Ginzburg–Landau simulations

In the time-dependent Ginzburg–Landau simulations based on Nakanishi’s model^78^ as discussed in the main text, we describe the physical CDW modulation *ρ*_*l*_ in layer *l* of the material as given in equation (2). This notation effectively strips the equilibrium in-plane CDW periodicity **q**_*j*_ from the additional out-of-equilibrium modulation given by the three coupled order parameters *ϕ*_*i*,*l*_. Numerically integrating the equation of motion for the phenomenological free energy *F* of the system, as in

4 $$\frac{\updelta {\phi }_{j,l}}{\updelta t}=-{{\varGamma }}\frac{\updelta F}{\updelta {\phi }_{j,l}^{* }}$$

where Φ^*^_jl_ denotes the complex conjugate of *Φ*_*j*__*l*_, then yields the spatiotemporal dynamics shown in Fig. 4 and in Supplementary Video 5. The parameter *Γ* controls the overall timescale of the dynamics. For the free-energy functional, we choose an approach tailored to model the phase diagram of the different three-dimensional CDW configurations in 1T-TaS_2_ (ref. ^78^), that is

5 $$\begin{array}{lll}F&=&\mathop{\sum}\limits_{l}\displaystyle\int{\rm{d}}^{2}r\left[\mathop{\sum}\limits_{j}\left({\phi }_{j,l}^{* }{A}_{j}({\bf{q}}_{j}-i\nabla ){\phi }_{j,l}+B{\left\vert {\phi }_{j,l}\right\vert }^{4}\right.\right.\\ &+&\left.C{\left\vert {\phi }_{j,l}{\phi }_{j+1,l}\right\vert }^{2}+E\,{{{\rm{Re}}}}({\phi }_{j,l}^{3}{\phi }_{j+1,l}^{* })\right)\\ &+&\left.D\,{{{\rm{Re}}}}({\phi }_{1,l}{\phi }_{2,l}{\phi }_{3,l})\right]\\ &+&\mathop{\sum}\limits_{l}G\displaystyle\int{{{{\rm{d}}}}}^{2}r\mathop{\sum}\limits_{j}\,{{{\rm{Re}}}}\left[{{\mathrm{e}}}^{i{g}_{1}}{\phi }_{j,l}^{* }{\phi }_{j,l+1}\right.\\ &+&\left.a{{\mathrm{e}}}^{i{g}_{2}}{\phi }_{j,l}^{* }{\phi }_{j,l+2}\right].\end{array}$$

Therein, energy minimization of the *B* term and the *C* term (referred to as nonlinear terms in the main text) ensures a local equilibration of the CDW amplitude, the phasing term *D* establishes relative phase relations between the order parameters that favour well-defined CDW maxima in a hexagonal arrangement (Supplementary Fig. 2), and *∇* denotes the nabla operator. The transition from a local lock-in of the CDW with the underlying main lattice in the low-temperature, commensurate phase of 1T-TaS_2_ to the incommensurate modulations found up to a critical temperature *T*_C_ = 1 is governed by a temperature-dependent competition between the commensurability energy *E* and the kinetic energy *A* (also the gradient term). To account for the temperature-dependent relative orientation between the NC wave vector and the main lattice periodicities^74^, the kinetic energy includes a softness towards a distortion of the order parameter along the azimuthal component. One finds^78^

6 $${A}_{j}\left({\bf{q}}_{j},T\right)=T-{T}_{{\mathrm{C}}}+(1-{\xi }_{j})u+{\xi }_{j}v(1-\cos 6{\varphi }_{j})$$

with

7 $${\xi }_{j}=1-s{(| {\bf{q}}| -| {\bf{q}}_{j}| )}^{2}/| {\bf{b}}_{j}{| }^{2}$$

where *φ*_*j*_ describes the angle between the wave vectors **q** and **q**_*j*_; **b**_*j*_ are the reciprocal lattice vectors of the undistorted structure; and *u*, *v* and *s* are parameters.

Along the out-of-plane components, equation (5) perturbationally treats the coupling of the individual layers to their nearest neighbouring and next-nearest neighbouring layers via the parameters *G* and *a*, respectively, while the finite phase factors *g*_1_ and *g*_2_ ensure the establishment of the expected stacking periodicity^78^.

We find that a dimensionless parameter set with *B* = 5 × 10^−4^, *C* = 1 × 10^−3^, *D* = −5 × 10^−4^, *E* = −7.5 × 10^−6^, *T* = 0.975, *s* = 6, *u* = 1.3, *v* = 0.4, *G* = 0.4, *a* = 0.5, *g*_1_ = −0.7, *g*_2_ = 0.7 and *Γ* = 1.43 × 10^−2^ reproduces our experimental results. Our simulation volume consists of 51 individual layers with approximately 250 × 290 CDW unit cells along each lateral dimension.

The corresponding CDW phase patterns are shown in Fig. 4f,g, as well as Supplementary Video 5, where we plot the phase of one of the order parameters Im(*ϕ*_3,*l*_). In this representation, the lowest-energy configuration, that is, a long-range-ordered CDW, corresponds to a flat phase profile within the layers and relative phase shifts of 2π/3 between them. Given the unknown configuration of defects directly after the optical excitation, we choose a stack of uncorrelated layers where both amplitude and phase of the *ϕ*_*j*,*l*_ values are randomized as starting conditions, accounting for the negligible influence of the initial state on the simulated dynamics while avoiding unnecessary complexity of the model. In this out-of-equilibrium setting, the few-picosecond build-up of CDW amplitude observed in the experiments is mediated by the nonlinearities *B* and *C*, resulting in the dense network of phase singularities described in the main text. As a consequence, translational correlations are short range, but the notion of sixfold orientational symmetry is weakly preserved. The subsequent defect kinetics are determined by the gradient term *A* and the perturbational consideration of the interlayer coupling *G*, approaching a three-dimensional, long-range-ordered state via defect recombination.

To extract spot widths from the simulation, we perform a three-dimensional Fourier transformation of the *ϕ*_*j*_ values at representative stages of the temporal dynamics to derive the simulated reciprocal lattice rod. Summing the squared modulus of the momentum distribution in reciprocal lattice planes corresponding to the out-of-plane momentum |***b***_3_*| (considering the different rotations of the respective **q**_*j*_) for all three *ϕ*_*j*_ values then gives the simulation analogue to the experimental diffraction spots depicted in Fig. 3b. The elastic properties of the CDW defined by the parameters *u* and *v* result in a larger broadening along the azimuthal axis than along the radial axis throughout the entire dynamics. For high defect densities, the spot width ratio is additionally enhanced, reproducing the expected influence of interacting dislocations on the orientational correlation function^58^. Fitting a two-dimensional Lorentzian function to this in-plane spot profile yields the corresponding spot widths *γ*_sim_. Along the out-of-plane directions, we assign the integrated intensity of the CDW diffraction spot in every reciprocal lattice plane to its out-of-plane component and fit a Gaussian function centred at the corresponding equilibrium stacking periodicities |***b***_3_*| to the resulting one-dimensional intensity distribution. The derived spot widths *γ* (full-width at half-maximum) over the course of the dynamics are displayed in Fig. 4a. For adequate comparison between simulation and experimental results, we include the instrument resolution *γ*_r_ in the form $\gamma =\sqrt{{\gamma }_{{{{\rm{sim}}}}}^{2}+{\gamma }_{{\mathrm{r}}}^{2}}$, where *γ*_r_ is the corresponding spot width measured at late times.

Within this model, the phase formation is governed by the kinetics of point-like defects that appear as vortices in the *ϕ*_*j*,*l*_. For the ‘flux line’ length shown in Fig. 4d, we assume vortices in neighbouring layers to be correlated when their spatial separation along the in-plane coordinates is less than five CDW lattice vectors. At early times, this definition of the interlayer vortex correlation is additionally influenced by coincidental alignment stemming from the high initial density of defects within every layer. We correct for this influence by subtracting the temporal evolution of the flux line length derived in the same manner for the case of uncoupled CDW layers.

The average energy of an individual vortex for both the coupled and uncorrelated stack of CDW layers is derived by integrating equation (5) at every step of the dynamics and by additionally considering the ground-state energy of the equivalent fully equilibrated system. For the latter, we chose a long-range-ordered CDW phase in the final stacking configuration as the initial condition and let the local amplitude relax until the system reached its energy minimum.

### Reporting summary

Further information on research design is available in the Nature Portfolio Reporting Summary linked to this article.

## Online content

Any methods, additional references, Nature Portfolio reporting summaries, source data, extended data, supplementary information, acknowledgements, peer review information; details of author contributions and competing interests; and statements of data and code availability are available at 10.1038/s41563-023-01600-6.

### Supplementary information

Supplementary Information Supplementary Figs. 1–3, Notes 1 (dynamical electron diffraction simulations) and 2 (fitting of specimen tilt angles) and captions for Videos 1–5.

Reporting Summary

Supplementary Video 1 Electron beam tilt series around the ZOLZ. Tilting the electron beam around the ZOLZ modulates diffracted intensities. For higher beam tilts, first-order CDW reflections appear at larger wave vectors.

Supplementary Video 2 Electron beam tilt series around the FOLZ. Higher beam tilts shift the probing of first-order CDW reflections to smaller wave vectors.

Supplementary Video 3 Ultrafast electron diffraction of the NC-to-IC transition in 1T-TaS_2_. Video of the phase transition corresponding to the images displayed in Fig. 3b, recorded with a laser pump fluence of 3.7 mJ cm^–2^ and an electron spot diameter of 1.1 μm.

Supplementary Video 4 Ultrafast electron diffraction of the NC-to-IC transition in 1T-TaS_2_ in the FOLZ. Video of the phase transition averaged over several main lattice reflections in the FOLZ. Experimental parameters as in Supplementary Video 3.

Supplementary Video 5 Time-dependent Ginzburg–Landau simulations. Extended CDW phase modulation (left) and resulting simulated diffractogram (right) for one of the three order parameters in one of the simulated layers. The initial state exhibits a high density of point defects and a two-dimensional character. The system establishes interlayer correlations after around 10 ps, evident from the disappearance of three of the six CDW diffraction spots. More details on the simulations and the evaluations are given in the main text and in the Methods.

## Data Availability

The data presented in the Article and Supplementary Information are available from GRO.data (ref. ^79^).
