# Supplementary material for: Light-induced hexatic state in a layered quantum material
Source: Nat Mater. 2023 Jul 6;22(11):1345–51. doi: 10.1038/s41563-023-01600-6 (PMC10627829; doi:10.1038/s41563-023-01600-6)
Supplement: Supplementary file 1 — Supplementary Figs. 1–3, Notes 1 (dynamical electron diffraction simulations) and 2 (fitting of specimen tilt angles) and captions for Videos 1–5. [file 41563_2023_1600_MOESM1_ESM.pdf]

nature materials

Article

https://doi.org/10.1038/s41563-023-01600-6

Light-induced hexatic state in a layered quantum material

In the format provided by the authors and unedited

Contents

Supplementary Note 1: Dynamical electron diffraction simulations 2

Supplementary Note 2: Fitting of specimen tilt angles 3

Supplementary Video S1: Electron beam tilt-series around the ZOLZ 4

Supplementary Video S2: Electron beam tilt-series around the FOLZ 4

Supplementary Video S3: Ultrafast electron diffraction of the NC-to-IC transition in $1T$-TaS_2 4

Supplementary Video S4: Ultrafast electron diffraction of the NC-to-IC transition in $1T$-TaS_2 in the FOLZ 4

Supplementary Video S5: Time-dependent Ginzburg-Landau simulations 4

Supplementary Figure S1: Fitting of specimen tilt angle and reconstructed rocking curves 5

Supplementary Figure S2: CDW formation in time-dependent Ginzburg-Landau simulations 6

Supplementary Figure S3: Real-space and reciprocal-space structures of CDW phases in $1T$-TaS_2 7

Supplementary Note 1: Dynamical electron diffraction simulations

Within a single diffractogram, scattered CDW intensities depend on the excitation error, i.e., the mismatch to an exactly fulfilled Laue condition, that is determined by the relative tilt angle between specimen and electron beam. In order to determine this tilt angle from measured intensities in our tilt-series, we perform dynamical diffraction simulations based on the scattering matrix approach. Therein, the incident electron wave function inside the crystal is divided into individual beams n scattered by a respective crystal periodicity $\vec{k}_n$[1]. The beam amplitude Ψ_n as a function of the penetration depth z can then be described as

$$\frac{\mathrm{d}\Psi_n}{\mathrm{d}z} = i\pi \sum_{n'=1}^{N} \left[2\, s_{n'}\, \delta\left(n-n'\right) + \xi_{n,n'}\right] \Psi_{n'}\,. \tag{S1}$$

Equation S1 is the *Darwin-Howie-Whelan equation* for elastic scattering of high-energy electrons. It can be expressed in terms of a scattering matrix S_N and then integrated numerically in close analogy to the treatment of N coupled harmonic oscillators[2]. The diagonal elements of this matrix account for the scattering geometry in the form of the excitation error s_n. The transfer of scattering amplitude between beams n and n' is mediated by the off-diagonal elements $\xi_{n,n'}$ representing the effective scattering length, thereby incorporating the influence of the specimen. Specifically, $\frac{1}{\xi_{\vec{k}}} \propto \lambda|V_{\vec{k}}|$ where λ is the electron wavelength and $V_{\vec{k}}$ the Fourier component of the scattering crystal potential, i.e., the structure factor associated with the wave vector $\vec{k}$.

Our simulations are based on the reported crystal structure of the NC phase[3] (Fig. S3a) where we approximate the incommensurate lattice distortion by a supercell comprised of $45 \times 45 \times 3$ unit cells of the undistorted structure (see ref.[4] for a justification of this approximation), while the out-of-plane periodicity of the NC phase is given by the $\langle\sigma_{h2}\rangle$ stacking described in detail in ref.[5]. The structure factor of the material is calculated with the atomic scattering factors for tantalum and sulphur[6] listed in table I, yielding scattering lengths of, e.g., $\xi = 108\,\mathrm{nm}$ for a scattering vector relating a first-order main lattice reflection to the direct beam, and $\xi = 1545\,\mathrm{nm}$ for a first-order NC spot relative to its associated main lattice spot.

In our simulations, we consider 61 main lattice reflections situated in the ZOLZ, in addition to a total of 786 CDW spots of first and second-order in the FOLZ and in the ZOLZ, respectively. As such, only those wave vectors yielding a scattering strength of more than 2 % of the maximum found at $\vec{q} = 0$ are taken into account. Furthermore, we include the effects of finite temperatures on scattered intensities in the form of the reported Debye-Waller factors of tantalum and sulphur at room-temperature[7]. A numerical integration of equation S1 with varying sample thickness and electron beam incidence then allows us to fit the overall specimen tilt following the approach described in the subsequent section.

A resulting simulated diffractogram for an exemplary beam incidence from our measurements is depicted in Fig. S1b. We account for a spatially varying sample orientation and the finite electron beam convergence angle by averaging over beam tilts within a range of 1.5 mrad centred around the fitted beam tilt. Since electron diffractograms, especially those recorded close to zone-axis orientation, always feature multiple diffraction spots of significant intensity, the average 70 nm thickness of our sample results in non-negligible effects of dynamical scattering on the measured intensities, leading to pronounced deviations from kinematical scattering theory. In particular, diffraction spots of the undistorted lattice situated in the vicinity of the Laue circle in the ZOLZ display strong intensity oscillations during a (virtual) beam tilt series, and, additionally, are highly susceptible to small variations of the tilt direction (dotted rocking curves in Fig. S1c). In contrast, first-order CDW reflections found at larger wave vectors are less affected by multiple

	i	a_i [Å][6]	b_i [Å^2][6]	W[Å^2][7]
Ta	1	0.3835	0.0810	0.1
	2	1.6747	0.8020	
	3	3.2986	4.3545	
	4	4.0462	19.9644	
	5	3.4303	73.6337	
S	1	0.0915	0.0838	2
	2	0.4312	0.7788	
	3	1.0847	4.3462	
	4	2.4671	15.5846	
	5	1.0852	44.6365	

Table I Parameters used in the diffraction simulation. The atomic scattering factors f of tantalum and sulphur to calculate the structure factor for scattering by a wave vector with magnitude k are derived following $f = \sum_i a_i \exp\left(-b_i k^2\right)$. The coefficients apply to $k < 6/\text{Å}$. The structure factor is additionally modulated by the Debye-Waller factor $\exp\left(-2Wk^2\right)$, the specified values for W apply to a broad temperature range around $\approx 300\,\mathrm{K}$.

scattering events (Fig. S1g). For these spots, the simulations yield only minor intensity modulations along k_z and a small shift of the rod centre.

Supplementary Note 2: Fitting of specimen tilt angles

In any TEM, the electron beam angle of incidence can be set by beam deflectors, the resulting shift of the diffractogram in the detector plane yields a reliable measure of the relative beam tilt between two settings. The absolute beam tilt, however, is encoded in diffracted intensities and requires fitting routines based on diffraction simulations[8].

Using the simulation approach outlined in the previous section, we calculated more than 32 000 diffractograms with variable angles of incidence, while averaging over the thickness distribution of our specimen (see Supplementary Material of ref.[9]) and a relative angle of incidence between specimen and electron beam of 1.5 mrad, considering both the electron beam convergence angle as well as spatial variations of the sample orientation. The simulated intensity $T_{i,j}$ of a diffraction spot j for every incidence i is then compared to the experimental intensity P_j measured before time-zero.

The strong impact of dynamical scattering leads to ambiguous fitting results for the individual T_i. To overcome this limitation, we additionally make use of the known relative beam tilts Δ in our tilt series, consisting of delay scans recorded under eight different angles of incidence. Considering the intensities of all main lattice and first-order CDW reflections, this approach yields the image correlation index[8]

$$Q_i = \prod_{\Delta=1}^{8} \frac{\sum_j^m P_j(\Delta)\, T_{i,j}(\Delta)}{\sqrt{\sum_j^m P_j^2(\Delta)}\sqrt{\sum_j^m T_{i,j}^2(\Delta)}}, \tag{S2}$$

where $Q_i = 1$ indicates a perfect agreement between experiments and simulation.

The calculated Q_i are depicted in Fig. S1h. We derive a maximum correlation index of $Q_i = 0.89$. The corresponding beam tilt results in the simulated intensities of main lattice spots shown in Fig. S1c and yields rocking curves for first-order CDW reflections that are centred around the wave vector of the equilibrium CDW stacking sequence (Fig. S1d-f, see also the reference tilt-series in Supplementary Videos S1 and S2, and Fig. S3d-e).

References

[1] De Graef, M. *Introduction to Conventional Transmission Electron Microscopy* (Cambridge University Press, Cambridge, 2003).

[2] Fujimoto, F. Dynamical Theory of Electron Diffraction in Laue-Case, I. General Theory. *Journal of the Physical Society of Japan* **14**, 1558–1568 (1959).

[3] Spijkerman, A., de Boer, J. L., Meetsma, A., Wiegers, G. A. & van Smaalen, S. X-ray crystal-structure refinement of the nearly commensurate phase of $1T$-TaS_2 in (3+2)-dimensional superspace. *Physical Review B* **56**, 13757–13767 (1997).

[4] Danz, T. C. *Ultrafast transmission electron microscopy of a structural phase transition.* Ph.D. thesis, Georg-August-Universität Göttingen, Göttingen (2021).

[5] Nakanishi, K. & Shiba, H. Theory of Three-Dimensional Orderings of Charge-Density Waves in $1T$-TaX_2 (X: S, Se). *Journal of the Physical Society of Japan* **53**, 1103–1113 (1984).

[6] Colliex, C. *et al.* 4.3. Electron diffraction. In Prince, E. (ed.) *International Tables for Crystallography*, vol. C, 259–429 (International Union of Crystallography, 2006).

[7] Balaguru Rayappan, J. B., Raj, S. A. C. & Lawrence, N. Thermal properties of $1T$-TaS_2 at the onset of charge density wave states. *Physica B: Condensed Matter* **405**, 3172–3175 (2010).

[8] Rauch, E. F. & Véron, M. Automated crystal orientation and phase mapping in TEM. *Materials Characterization* **98**, 1–9 (2014).

[9] Danz, T., Domröse, T. & Ropers, C. Ultrafast nanoimaging of the order parameter in a structural phase transition. *Science* **371**, 371–374 (2021).

Supplementary Video S1: Electron beam tilt-series around the ZOLZ. Tilting the electron beam around the ZOLZ modulates diffracted intensities. For higher beam tilts, first-order CDW reflections appear at larger wave vectors.

Supplementary Video S2: Electron beam tilt-series around the FOLZ. Higher beam tilts shift the probing of first-order CDW reflections to smaller wave vectors.

Supplementary Video S3: Ultrafast electron diffraction of the NC-to-IC transition in $1T$-TaS_2. Movie of the phase transition corresponding to the images displayed in Fig. 3b, recorded with a laser pump fluence of $3.7\,mJ/cm^2$ and an electron spot diameter of $1.1\,\mu m$.

Supplementary Video S4: Ultrafast electron diffraction of the NC-to-IC transition in $1T$-TaS_2 in the FOLZ. Movie of the phase transition averaged over several main lattice reflections in the FOLZ. Experimental parameters as in Supplementary Video S3.

Supplementary Video S5: Time-dependent Ginzburg-Landau simulations. Extended CDW phase modulation (left) and resulting simulated diffractogram (right) for one of the three order parameters in one of the simulated layers. The initial state exhibits a high density of point defects and a two-dimensional character. The system establishes interlayer correlations after around 10 ps, evident from the disappearance of three of the six CDW diffraction spots. More details on the simulations and the evaluations are given in the main text and in the Method section.

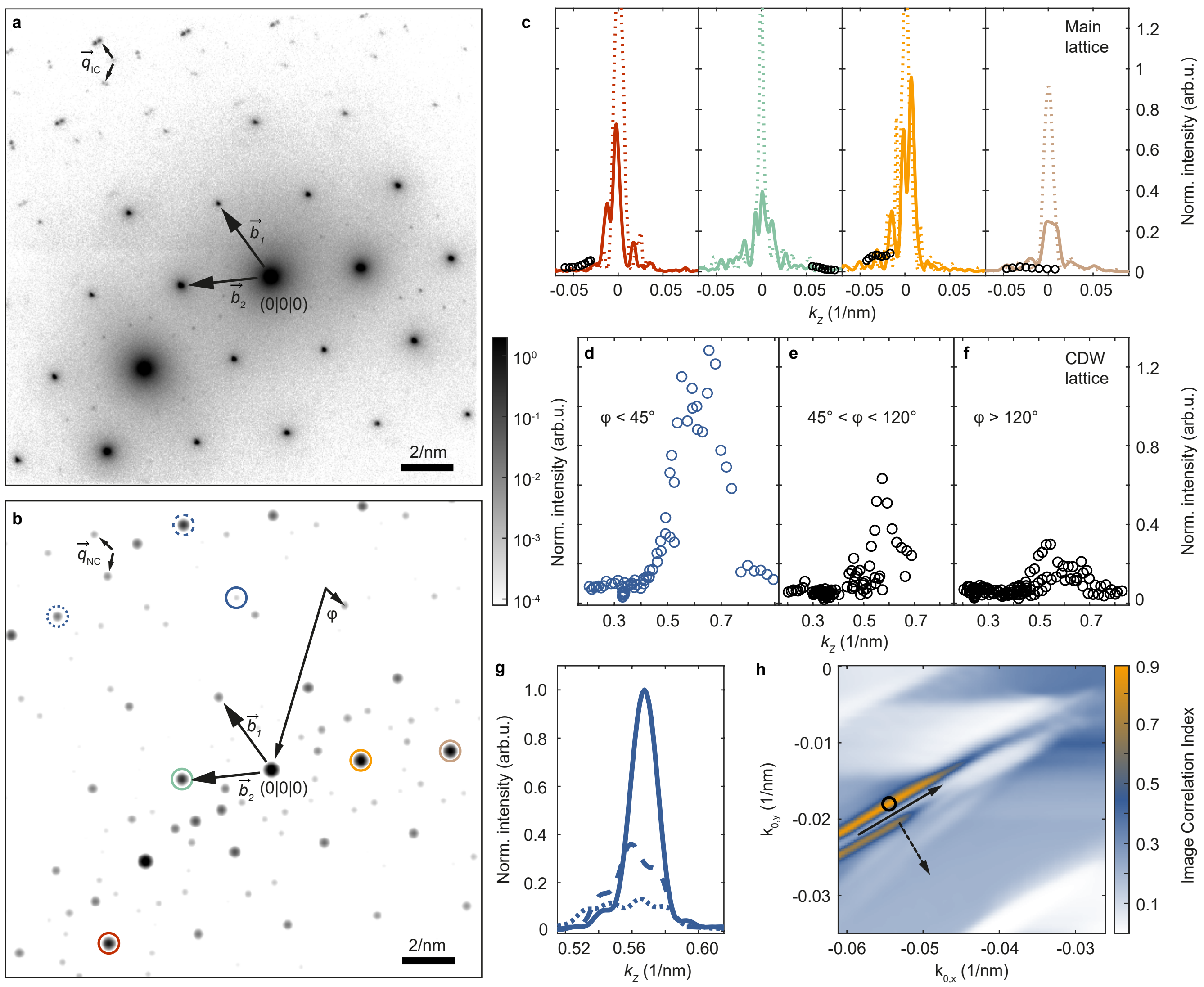

Supplementary Fig. S1 Fitting of specimen tilt angle and reconstructed rocking curves. **a** Exemplary electron diffractogram recorded with one of the overall eight different specimen tilt angles used to reconstruct CDW rocking curves. The displayed image is a temporal average over all stages of the phase formation kinetics as can be recognised from the presence of spots corresponding to both CDW phases. Direct electron detection enables simultaneous recording of scattered intensities spanning five orders of magnitude (see intensity scale). **b** Simulated electron diffractogram for the NC phase of 1*T*-TaS_2 for the fitted tilt angle in **a**. Coloured circles mark the spots belonging to the measured and simulated rocking curves in **c** and **g**, respectively. The scattered CDW intensity is additionally modulated by the CDW structure factor which predominantly depends on the angle φ (see Methods). **c** Measured (black circles) and simulated (solid lines) rocking curves of the main lattice reflections indicated in **b**. The dotted curves describe the rocking curve for an additional sample tilt of 0.29° along the direction indicated by the dotted arrow in **h**, exemplifying the pronounced influence of dynamical scattering on bright intensity reflections situated in the ZOLZ. Measured data is rescaled by a global factor for all curves to match the simulated intensities. **d**-**f** Measured NC CDW rocking curves for the fitted specimen tilt angle and sorted by the CDW structure factor (see Methods). The bright-intensity CDW spots in **d** are used to extract the out-of-plane shape of the reciprocal lattice rod discussed in the main text. Especially CDW spots with smaller structure factor (**e** and **f**) are subject to dynamical scattering. **g** Simulated CDW rocking curves for the spots highlighted in **b**. Overall, dynamical scattering has a smaller influence on first order CDW intensities than on main lattice reflections in the ZOLZ. Still, it causes an additional broadening of the spot profile and a shift of the k_z-component with highest CDW intensity (dotted curves). **h** Fitted quality factors of the template matching described in Supplementary Note 2 as a function of the k_x, k_y-components of the normalised incident wave vector k_0. The black circle indicates the fitting results, the tilt direction for the rocking curves in **c** and **g** is given by the solid black arrow. A similar virtual tilt series only shifted by 0.29° along the direction given by the dotted arrow yields the dotted rocking curves displayed in **c**.

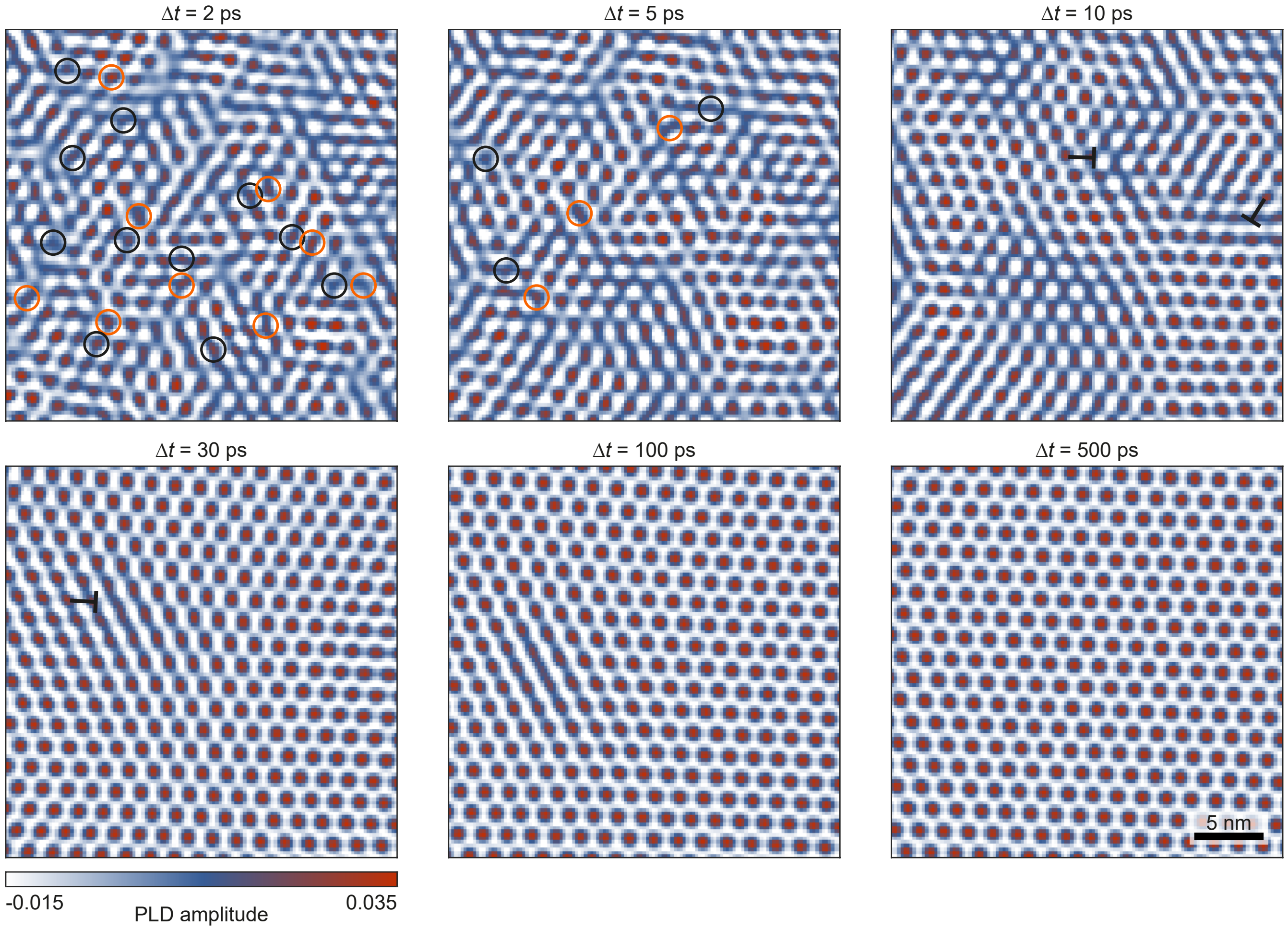

Supplementary Fig. S2 CDW formation in time-dependent Ginzburg-Landau simulations. CDW pattern ρ_l (see main text) at variable temporal delay derived from the time-dependent Ginzburg-Landau simulations. Orange and black circles highlight several disclinations with seven-fold and five-fold coordination, respectively (2 ps and 5 ps temporal delay). The late stage dynamics is governed by the annihilation of dislocations (black markers at 10 ps and 30 ps temporal delay).

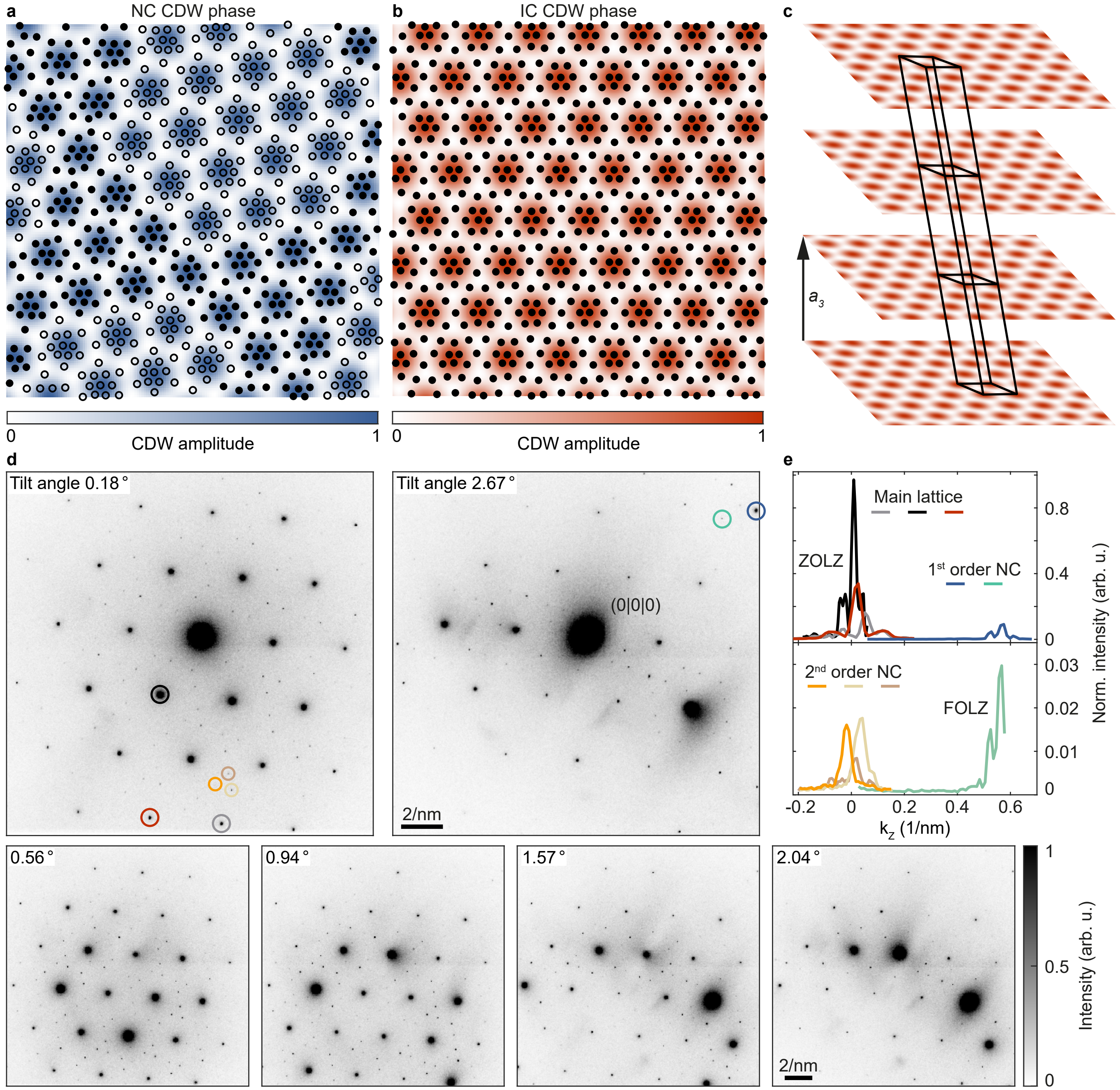

Supplementary Fig. S3 Real-space and reciprocal-space structures of CDW phases in 1*T*-TaS_2 **a** The charge density modulation of the NC CDW (blue) is characterised by significant contributions from higher harmonics of the modulation wave vector, resulting in a domain-like atomic configuration (black circles, distortions exaggerated five times for better visibility). Regions where the PLD is close to the commensurate modification (open circles) are separated by domain walls resembling the incommensurate type (filled circles). The displayed structure is based on the refinement in ref.[3]. **b** In the IC phase, the CDW (red) and the atomic displacements (filled circles) can be approximated by a single wave vector component (distortions exaggerated five times). **c** Along the out-of-plane direction $\vec{a_3}$, the CDW assumes a three-fold stacking in both phases, as exemplified here for the IC phase. Black lines represent the three-dimensional CDW unit cell. **d** Representative diffractograms of the NC phase from an electron-beam tilt-series (cf. also Supplementary Video S1). Coloured circles highlight the spots corresponding to the rocking curves in **e**. Under close-to-perpendicular illumination, the images contain spots in the zero-order Laue zone (ZOLZ), i.e., main lattice (bright intensity) and second order NC spots (low-intensity satellites, note also the intensity scale in **e**). Higher beam-tilts allow accessing first-order NC spots in the first-order Laue zone (FOLZ). The beam-tilt refinement is based on the dynamical electron diffraction simulation (supplementary Note 1) and the tilt-angle fitting described in Supplementary Note 2. The diffuse background in images recorded with higher beam-tilts is due to limitations imposed by aberrations in the alignment of the microscope's illumination system over the whole range of tilt angles. **e** Exemplary rocking curves for main lattice reflections (grey, black, red) and second-order NC spots (orange, beige, brown) in the ZOLZ, and for first-order NC spots in the FOLZ (blue, green). Particularly reflections located in the ZOLZ are subject to dynamical scattering, leading to additional intensity modulations and shifts of the maximum position along the out-of-plane momentum k_z.
